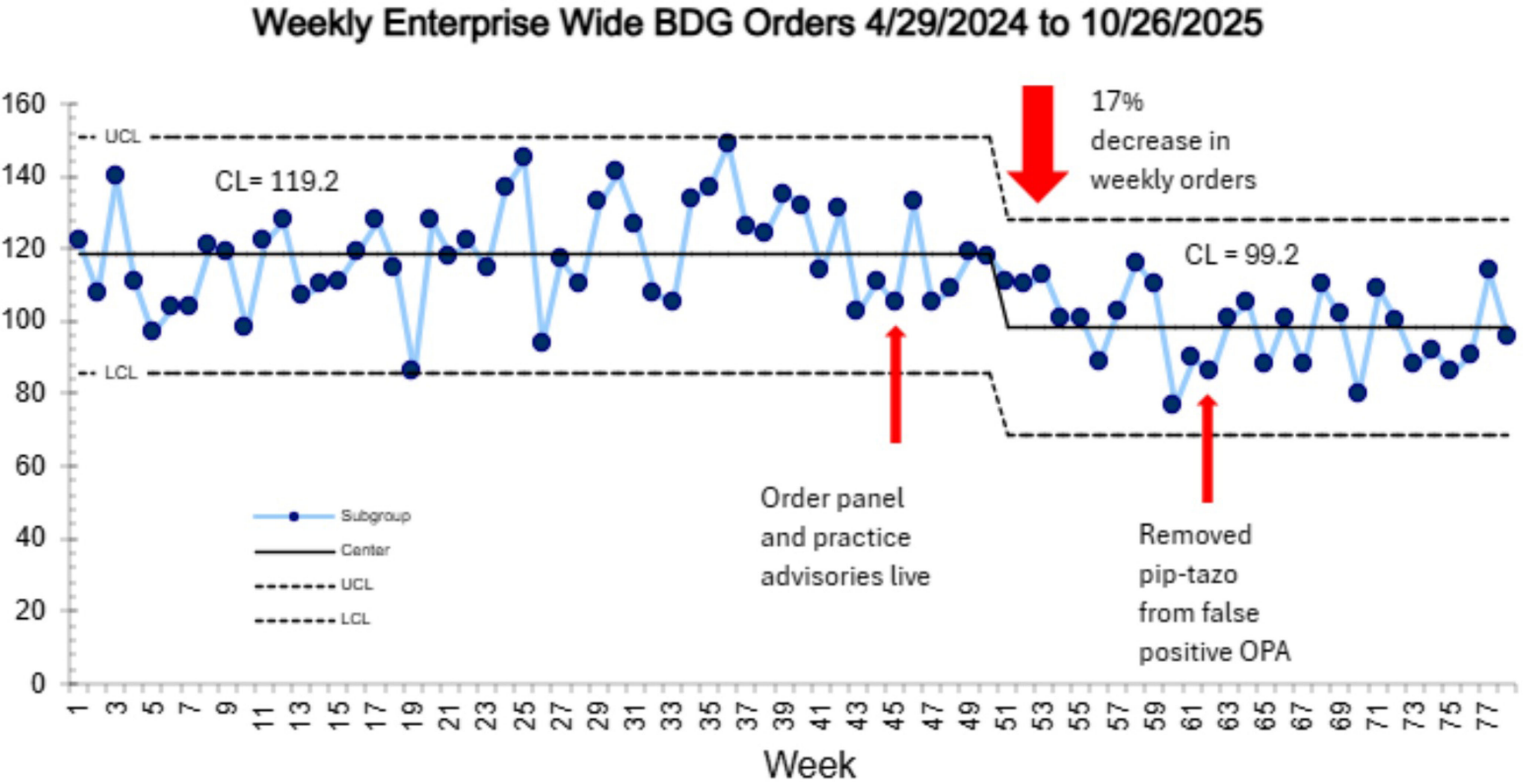# 183 Hub to Heart: Reducing CLABSIs in High-risk Pediatric Population Through a Back-to-Basics Competency to Support Evidence-based Practice

**DOI:** 10.1017/ash.2026.10577

**Published:** 2026-06-23

**Authors:** Emi Hayashi, Harjot Singh, Regina Wulff, Christine J. Kubin, E. Yoko Furuya, Matthew Simon, Nishant Prasad, Robin Goldberg, Thomas Rush, Harold Horowitz, Jamie Marino

**Affiliations:** 1 New York Presbyterian Columbia University Irving Medical Center; 2 Weill Cornell Medical College; 3 New York Presbyterian; 4 New York Presbyterian Hospital - CUMC; 5 Columbia University; 6 NewYork-Presbyterian Queens; 7 Division of Infectious Diseases, Department of Medicine, NewYork-Presbyterian Brooklyn Methodist Hospital; 8 Weill Cornell Medicine

## Abstract

**Background:** Beta-D-glucan (BDG) is a blood test that supports invasive fungal infection diagnoses, but is often over-ordered approximately 50% of the time, particularly in low-risk patients or for fungi not associated with BDG production. High rates of inappropriate use and frequent false positives, often driven by certain clinical scenarios or medication interactions, can lead to repeat testing and unnecessary procedures and treatment. While audit-and-feedback interventions can reduce test misuse, they are not feasible or sustainable for most hospitals. We implemented diagnostic stewardship of BDG through a guideline-based order panel with embedded decision support and interruptive alerts to replace a standalone test (figure 1). This quality improvement (QI) study aimed to reduce the use of BDG in the adult inpatient setting by 20% by 3/2026. **Methods:** From 3/5 -10/25/25, a multi-disciplinary team implemented this QI project at 8 campuses using iterative Plan-Do-Study-Act cycles. The primary outcome was the number of weekly BDG tests ordered across all campuses. Process measures included the number of interruptive alerts, the rate of duplicate tests, and adherence to order panel recommendations regarding appropriate host factors for BDG testing. Data was collected via the electronic health record. Statistical process charts (SPC) were used to display and analyze data, and Associate Process Improvement rules were applied to detect special cause variation. **Results:** Across the health system, the average weekly serum BDG orders decreased by 17% (figure 2). The false-positive and repeat-test alerts fired on average 76 and 8 times/week, respectively. Due to frequent false-positive alerts, low-impact factors (e.g., piperacillin-tazobactam) were removed, reducing average weekly false positive alerts from 101 in the first half of the intervention to 52. 77% of the false positive alerts were among patients on internal medicine, critical care, and oncology services. Based on chart review of 50 medicine, critical care, and oncology patients, approximately 51% of false-positive OPAs led to successful test interruption (i.e. delayed BDG ordering by ≥6 hours). The percentage of weekly duplicate tests decreased from 23% pre-intervention to 20% during the intervention. Accurate adherence to the order panel in this subset was 76%. **Conclusion:** In this study, we successfully implemented scalable BDG diagnostic stewardship, achieving a sustained 17% reduction in weekly BDG orders and nearly reaching the 20% target after 32 weeks. Further optimization of OPAs, along with provider feedback and education, may yield additional improvements.

**Figure:**
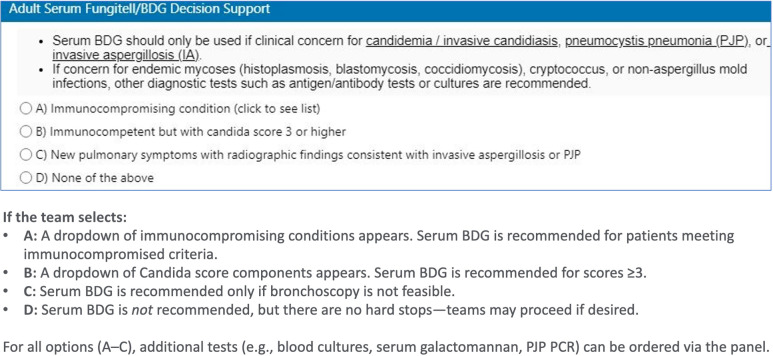

**Figure:**
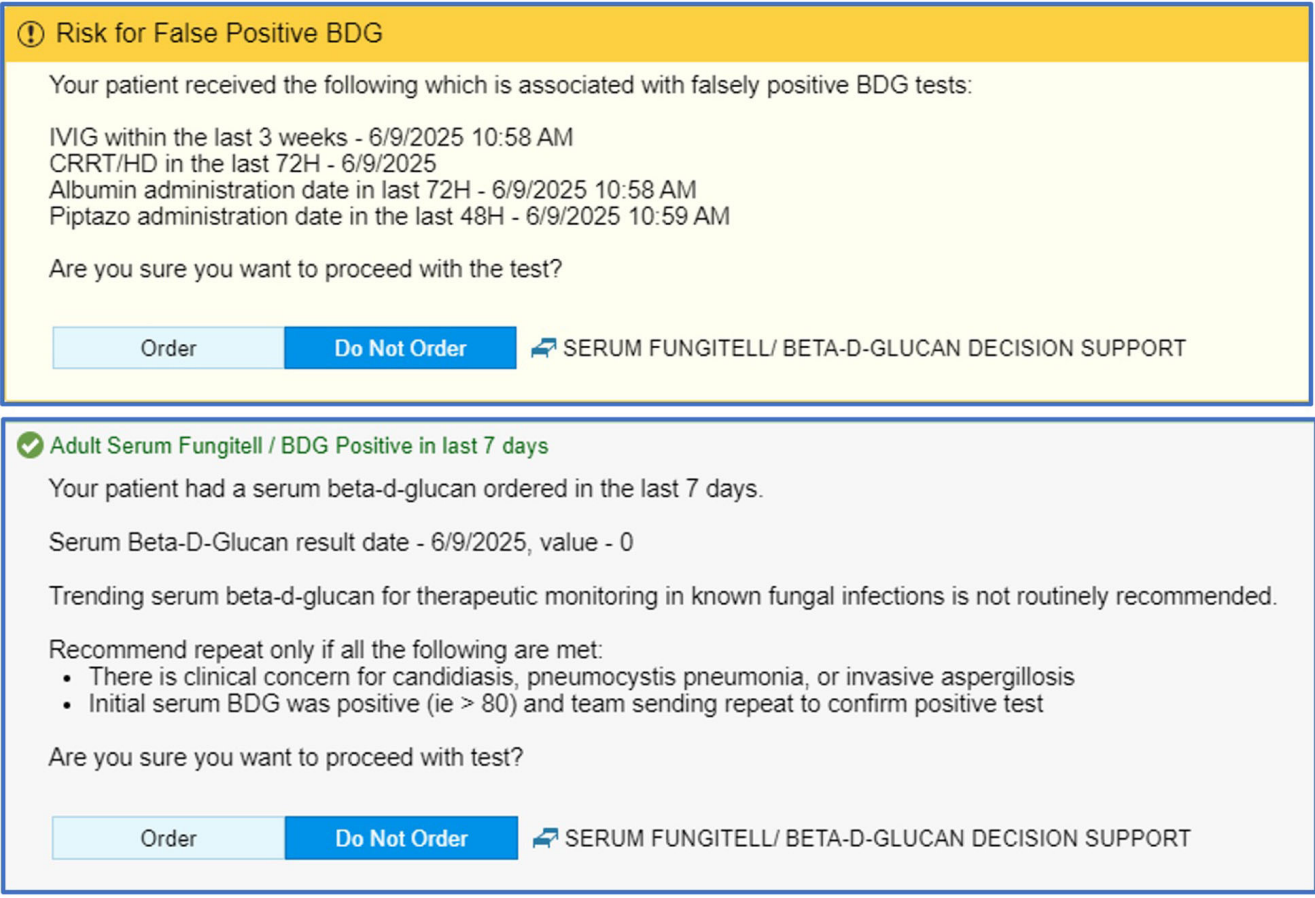

**Figure:**